# Adult cancer patients’ barriers to and satisfaction with care at a National Cancer Hospital in Vietnam

**DOI:** 10.1371/journal.pone.0303157

**Published:** 2024-05-09

**Authors:** Thinh Toan Vu, Marina Weiss, Linh Thi-Hoai Nguyen, Huong Thanh Tran, Hien Thi Ho, Victoria Khanh Ngo

**Affiliations:** 1 Center for Innovation in Mental Health, Graduate School of Public Health & Health Policy, The City University of New York, New York, New York, United States of America; 2 Department of Community Health and Social Sciences, Graduate School of Public Health & Health Policy, The City University of New York, New York, New York, United States of America; 3 Asian University for Women, Chittagong, Bangladesh; 4 Vietnam National Cancer Institute, K Hospital, Hanoi, Vietnam; 5 Hanoi University of Public Health, Hanoi, Vietnam; Hosei University: Hosei Daigaku, JAPAN

## Abstract

**Study objective:**

This study assessed the overall satisfaction with oncological care, including barriers to care, and identified its associated predictors among adult cancer patients in Vietnam.

**Methods:**

In this cross-sectional study, we enrolled 300 adult cancer patients receiving inpatient care at a large urban oncological hospital between June and July 2022. Multivariable linear regression analyses examined associations between patient experiences and overall satisfaction ratings with cancer care.

**Results:**

The mean overall satisfaction with oncological care was 8.82 out of 10, with 98.0% recommending this facility to their friends and family. In an adjusted model, being female (β = 0.29, 95%CI: 0.04, 0.53), endorsing satisfaction with patient-nurse communication (β = 0.33, 95%CI: 0.13, 0.53), patient-doctor communication (β = 0.40, 95%CI: 0.11, 0.70), and psychoeducation about oncological medication management (β = 0.30, 95%CI: 0.14, 0.45) were positively associated with overall ratings. In contrast, individuals with delays in treatment scheduling reported lower overall satisfaction with oncological care (β = -0.38, 95%CI: -0.64, -0.13). Patients perceived health system, social/environmental, and individual barriers to care: worries about income loss due to attending treatment (43.3%); fear, depression, anxiety, and distress (36.8%); concerns about affordability of treatment (36.7%) and transportation problems (36.7%); and excessive waiting times for appointments (28.8%).

**Conclusion:**

This study showed high overall patient satisfaction with cancer care quality. Patient-centered communication strategies and psychoeducation about oncological medication management may be targeted to further enhance the cancer inpatient experience. Raising awareness about treatment options and services, and integrating mental health awareness into oncological care may ameliorate patient distress and facilitate greater satisfaction with oncological treatment processes.

## Introduction

Chronic diseases like cancer and cardiovascular diseases are a leading cause of death globally, accounting for 71.0% of global mortality each year, with even greater impacts in low- and middle-income countries (LMICs) [1]. Vietnam experienced a significant rise in new cancer diagnoses in 2020, with 182,563 cases reported [2], representing a threefold increase compared to 1990. The Vietnamese healthcare system, overburdened by rising cancer rates, is estimated to meet only 30–40% of the demand for cancer services due to limited resources [3]. Globally, the COVID-19 pandemic exacerbated care gaps, interrupting physical healthcare provision [4]. A Canadian population-based cohort study found a 14.1% reduction in cancer-related surgical procedures and a 20.7% reduction in radiation therapy during the COVID-19 pandemic, while cancer screenings decreased by 42.4% [5], but few datasets exist which explore this phenomenon in lower-income countries. Such care gaps decrease patients’ quality of life and health outcomes [6] as well as increasing caregiver burdens, and endangering caregivers’ health [6,7], suggesting that lack of adequate cancer treatment represents a significant obstacle not just to patients’ but also to their families’ and communities’ wellbeing. Therefore, identifying and addressing barriers to oncological treatment access at the individual, social, and system levels is imperative for patients and their communities. However, cancer inpatient satisfaction and barriers to oncological treatment during the COVID-19 pandemic in Vietnam are under-researched, particularly in inpatient care settings. Generating a locally contextualized evidence base of patient experience in cancer care is critical to better understanding patients’ unmet needs.

Contemporary best practices increasingly prioritize patient-centered care, which respects patient’s values and preferences, educates them about their conditions, ensures access to treatment, offers emotional support, involves loved ones, fosters a continuous care plan, provides physical comfort and coordinated care across providers [8]. This approach aims to improve patient satisfaction and augment patient ownership of and adherence to health-promoting lifestyle changes and prescribed treatments [9]. By gauging cancer patients’ satisfaction with their care journey, health care providers can identify areas for improvement, leading to better patients’ experiences and potentially even improved health outcomes. Previous studies among Vietnamese cancer patients primarily focused on health-related quality of life in both newly admitted and established cancer patients [10,11], with specific diagnoses such as gastric cancer [12] and breast cancer [13]. The healthcare facilities in Vietnam are divided into four levels, corresponding to a hierarchical administrative organization: communal, district, provincial, and national/central. However, only one study was conducted to evaluate inpatient experience at the provincial level and showed high patient satisfaction about the treatment experience with doctors and nurses, medical staff’s responses to the patients’ requests, explanation of the rationale for oncological medication management, and hospital environment (ranging from 87.8% to 100%) [14]. Less research, however, focused on inpatient satisfaction across cancer diagnoses specifically within tertiary-level oncological hospitals at the national level where the majority of cancer patients receive care.

This study aimed to address existing research gaps by examining the overall satisfaction with oncological care and its associated factors among adult cancer patients receiving care at Vietnam’s largest oncological hospital. To ensure a comprehensive understanding of cancer care, the study also explored the inpatient experience, including patient-health provider communication, physical environment, pain management, and medication management, and investigated the barriers that hindered patients from accessing high-quality cancer care.

## Materials and methods

### Study design and sample size

This cross-sectional study employed face-to-face surveys using the Research Electronic Data Capture at the largest specialized oncological hospital at the national level in Vietnam between June and July 2022. The methodology, including sample size calculation and sampling strategies, was described in our previous publication [15]. Particularly, we recruited cancer patients from six chemotherapy units and four radiotherapy units. Each unit was assigned a data collection period. If there were fewer than 30 patients, all were included; otherwise, a convenience sample was drawn for departments with more than 30 patients. Adults (≥18 years) diagnosed with cancer and undergoing treatment were eligible to participate [15]. A total of 300 patients were included in the analyses.

### Measurements

#### Satisfaction with oncological care and inpatient experience

This study used the Inpatient Assessment of Health Care (I-PAHC; 12 items) questionnaire [16] which assesses patient experience across five domains: communication with nurses (e.g., “*How often did nurses treat you with courtesy and respect*?*”*), communication with doctors (e.g., “*How often did doctors listen carefully to you*?*”*), physical environment (e.g., “*How often was the room you were sleeping in kept clean*?*”*), efficacy of pain management (e.g., “*How often was your pain well controlled*?*”*), and education about oncological medication management (e.g., “*How often did staff tell you what medicine was for*?*”*). Each question was scored from 1 (never) to 4 (always) with higher scores indicating better experience. The I-PAHC was validated in low-income and upper middle-income settings [16,17], and the Cronbach’s alpha in our study indicated good to excellent internal consistency (0.87). Additionally, patients were asked to rate their overall satisfaction with their cancer care from 0 to 10, with higher scores indicating higher satisfaction with service quality. Patients rated the likelihood to recommend the study hospital to their friends and family on a 4-point scale (definitely no, probably no, probably yes, and definitely yes). Responses were grouped into ‘definitely yes’ or ‘probably yes’ versus ‘definitely no’ or ‘probably no’.

#### Barriers to quality cancer care

The Barriers to Accessing Quality Health Care for Patients scale was used [18]. This 16-item questionnaire assesses barriers to healthcare across three domains: 1) challenges in healthcare system, 2) social/environment barriers, and 3) individual psychological, attitudinal, and behavioral barriers. Each item was scored on a 3-point scale from 0 (not a barrier) to 2 (a major barrier). “Somewhat of a barrier” or “a major barrier” were merged to report the percentage of having any barriers. We modified two items after the pilot study to best fit our study population and local context, and the Cronbach’s alpha showed good internal consistency (0.74).

#### Functional impairment

The 12-item World Health Organization Disability Assessment Schedule (WHODAS 2.0) was deployed to evaluate patients’ functioning, and corresponded to six domains: 1) life activities, 2) cognition, 3) self-care, 4) mobility, 5) getting along, and 6) participation [19]. Each item was rated on a scale from 1 (none) to 5 (extremely difficult), and a summary score (12–60) was calculated by summing all individual item score, with higher scores suggesting higher disability or loss of function. The Cronbach’s alpha in our cohort showed excellent internal consistency (0.93) [15].

#### Oncological characteristics

Data on patients’ cancer diagnoses and treatment was retrieved from medical records including primary cancer site, cancer metastatic stage, diagnosis date, treatment date, and oncological treatment methods. Additionally, patients were asked about immediate family members’ cancer history (yes vs. no), and the number of treatment visits to the study hospital [15].

*Sociodemographic characteristics* included age in years, gender (male vs. female), ethnicity (Kinh vs. other), education background (less than high school, high school, and college/university or higher), marital status (currently married vs. unmarried), current occupation (unemployed, retired, farmers, officers, etc.), monthly income among employed patients (in $), home environment (urban vs. rural), insurance status and coverage, and driving distance from home to the hospital (in miles) [15].

To ensure questionnaire validity, a comprehensive procedure was undertaken, as described elsewhere [15]. This involved a meticulous translation into Vietnamese and subsequent back-translation into English, conducted by two bilingual doctoral candidates. Expert input was sought for content validity, and pilot testing was conducted with a sample of 25 cancer inpatients.

### Data analysis

Data were cleaned daily to check the logic and analyzed using STATA (Version 17.0). All issues (e.g., response discrepancies) were raised and resolved on the same day as patient interviews. Frequency and percentage were used for demographic and oncological characteristics, and barriers to cancer care access. Mean and standard deviations (SD) were presented for inpatient experiences with service utilization, otherwise median and interquartile ranges (IQR) were reported because of normality violations (e.g., income and number of treatment visits at hospital). Variables that showed statistical significance in the bivariate analysis (t-test and one-way ANOVA, p<0.05), and supported by the literature review were included in the multivariable linear regression model to evaluate factors associated with the overall satisfaction with oncological care. The final model was evaluated based on (1) normality of residuals, (2) linearity relationship, (3) homoskedasticity, (4) multi-collinearity, and (5) other indices (e.g., adjusted R^2^).

### Ethical considerations

This study was approved by the Institutional Review Boards at the Hanoi University of Public Health, Hanoi, Vietnam (121/2022/YTCC-HD3). Written informed consent was obtained from all cancer patients included in this study.

## Results

### Demographic characteristics and its association with overall satisfaction with oncological care

Among 300 cancer patients, 52.3% were women and the mean age was 56 years (SD = 19.6) with 47.7% aged between 40 and 59. Approximately 60.0% of patients did not complete high school. The majority of participants were Kinh (88.0%) and married (91.7%). A third (32.7%) were farmers and 18.0% were retired. Approximately 20.0% self-reported being unemployed. Among employed patients, the mean monthly income was $173.9 (IQR: $65.2-$260.9). All participants were insured, and health insurance covered a mean of 86.8% of treatment expenses. More than two-thirds (69.0%) currently resided in rural areas and the median distance from their home to the hospital was 62.1 miles (IQR: 37.3–93.2 miles) (Table 1).

**Table 1:** Demographic characteristics of cancer patients in Vietnam: 2022.

	Total	Satisfaction with cancer care	p-value^a^
	N = 300	mean±SD	
**Age in years (mean±SD)**	56.0±19.6	-	
18–39	33 (11.0)	8.82±0.92	0.525
40–59	144 (48.0)	8.75±1.16	
60+	123 (41.0)	8.90±0.99	
**Gender**			<0.001
Male	143 (47.7)	8.53±1.18	
Female	157 (52.3)	9.08±0.88	
**Educational attainment**			0.131
Less than high school	179 (59.7)	8.75±1.17	
High school	83 (27.7)	8.84±0.89	
College/university or higher	38 (12.7)	9.13±0.81	
**Ethnicity**			0.207
Kinh	264 (88.0)	8.85±1.07	
Other	36 (12.0)	8.61±1.02	
**Current marital status**			0.064
Not married	25 (8.3)	9.20±0.87	
Married	275 (91.7)	8.79±1.08	
**Current employment**			0.119
Retired	54 (18.0)	8.81±0.80	
Unemployed	60 (20.0)	9.05±1.05	
Farmer	98 (32.7)	8.67±1.17	
Officer	39 (13.0)	8.95±1.02	
Business/service worker	27 (9.0)	8.48±1.21	
Skilled labor (seamstress, weaver)	12 (4.0)	9.17±1.03	
Housewife	10 (3.3)	9.10±0.88	
**Monthly income (Median (IQR)) ($)** ^b^	173.9 (65.2–260.9)	-	
**Home environment**			0.853
Rural	207 (69.0)	8.81±1.01	
Urban	93 (31.0)	8.84±1.19	
**Percentage of treatment expenses covered by health insurance**^c^ **(mean±SD)**	86.8±13.9	-	
**Distance from home to this facility (Median (IQR)) (miles)**	62.1 (37.3–93.2)	-	

^a^t-test and one-way ANOVA tests;

^b^$1 = 24,000 VNĐ;

^c^100% of patients had health insurance.

In the bivariate analyses, only gender was found to be associated with satisfaction with oncological care (p <0.001).

### Oncological characteristics and its association with overall satisfaction with oncological care

The treatment facility grouped patients by primary cancer site. The largest primary cancer site group was the head, neck, and lungs (representing 40.4% of patients), followed by gastrointestinal tract cancers and breast/gynecological cancers (30.3% and 29.3%, respectively) (Table 2). In terms of specific primary cancer origination sites, most patients (20.3%) were diagnosed with lung cancer followed by breast cancers (14.3%) and cervical and uterine cancers (9.3%). Stage III cancer was observed in 41.0% of participants, and a third (32.7%) were at stage IV. Over three-fourths of participants (78.0%) had been in treatment for less than a year. Only 19.0% of surveyed patients received their first treatment at this hospital. The median number of treatment visits was 5.0 (IQR: 2.0–12.5). Over a third of patients (36.4%) received combined chemotherapy and radiotherapy, while 48.3% and 15.3% received chemotherapy and radiotherapy aline, respectively. More than half (53.3%) also underwent surgery. About 30.0% of patients reported a positive immediate family history of cancer.

**Table 2:** Oncological characteristics among cancer patients in Vietnam: 2022.

	Total	Satisfaction with cancer care	p-value^a^
	N = 300	mean±SD	
**Cancer treatment group**			0.011
Head, neck, lungs	121 (40.4)	8.67±1.16	
Gastrointestinal tract	91 (30.3)	8.75±1.03	
Breast/gynecological	88 (29.3)	9.01±0.91	
**Cancer site**			0.002
Breast	43 (14.3)	8.95±0.90	
Lung	61 (20.3)	8.55±1.17	
Esophagus	25 (8.3)	8.44±1.16	
Cervical/uterine	28 (9.3)	9.11±0.92	
Nasopharynx	21 (7.0)	8.29±1.34	
Other	122 (40.7)	9.02±0.96	
**Metastatic stage**			0.60
Stage I	27 (9.0)	8.78±0.80	
Stage II	52 (17.3)	8.99±0.88	
Stage III	123 (41.0)	8.75±1.14	
Stage IV	98 (32.7)	8.83±1.12	
**Length of cancer treatment (mean±SD)**	3.8 (1.6–11.4)		0.039
<1 year	234 (78.0)	8.75±1.11	
≥1year	66 (22.0)	9.06±0.87	
**First treatment at this facility**			0.312
Yes	57 (19.0)	8.69±1.04	
No	243 (81.0)	8.85±1.07	
**Number of treatment visits at this facility (median (IQR))**	5.0 (2.0–12.5)	-	
**Oncological treatment**			0.088
Chemotherapy	145 (48.3)	8.92±1.05	
Radiation	46 (15.3)	8.92±0.87	
Both	109 (36.4)	8.64±1.15	
**Experiencing surgical treatment**			0.211
No	140 (46.7)	8.74±1.12	
Yes	160 (53.3)	8.89±1.01	
**Family history of cancer**			0.881
No	209 (69.7)	8.83±1.06	
Yes	91 (30.3)	8.81±1.07	
**Overall satisfaction with oncological care (0–10) (mean±SD)**	8.82±1.07	-	
0–6	8 (2.7)	-	
7–8	86 (28.7)	-	
9–10	206 (68.6)	-	
**Recommending the study hospital to friends and family**			0.004
No	6 (2.0)	7.58±1.74	
Yes	294 (98.0)	8.85±1.04	

^a^t-test and one-way ANOVA tests.

In the bivariate analyses, factors associated with satisfaction with oncological care included cancer treatment group, cancer site, and length of cancer treatment (all p-values <0.05).

### Overall satisfaction with oncological care and inpatient experience

**Fig 1:**
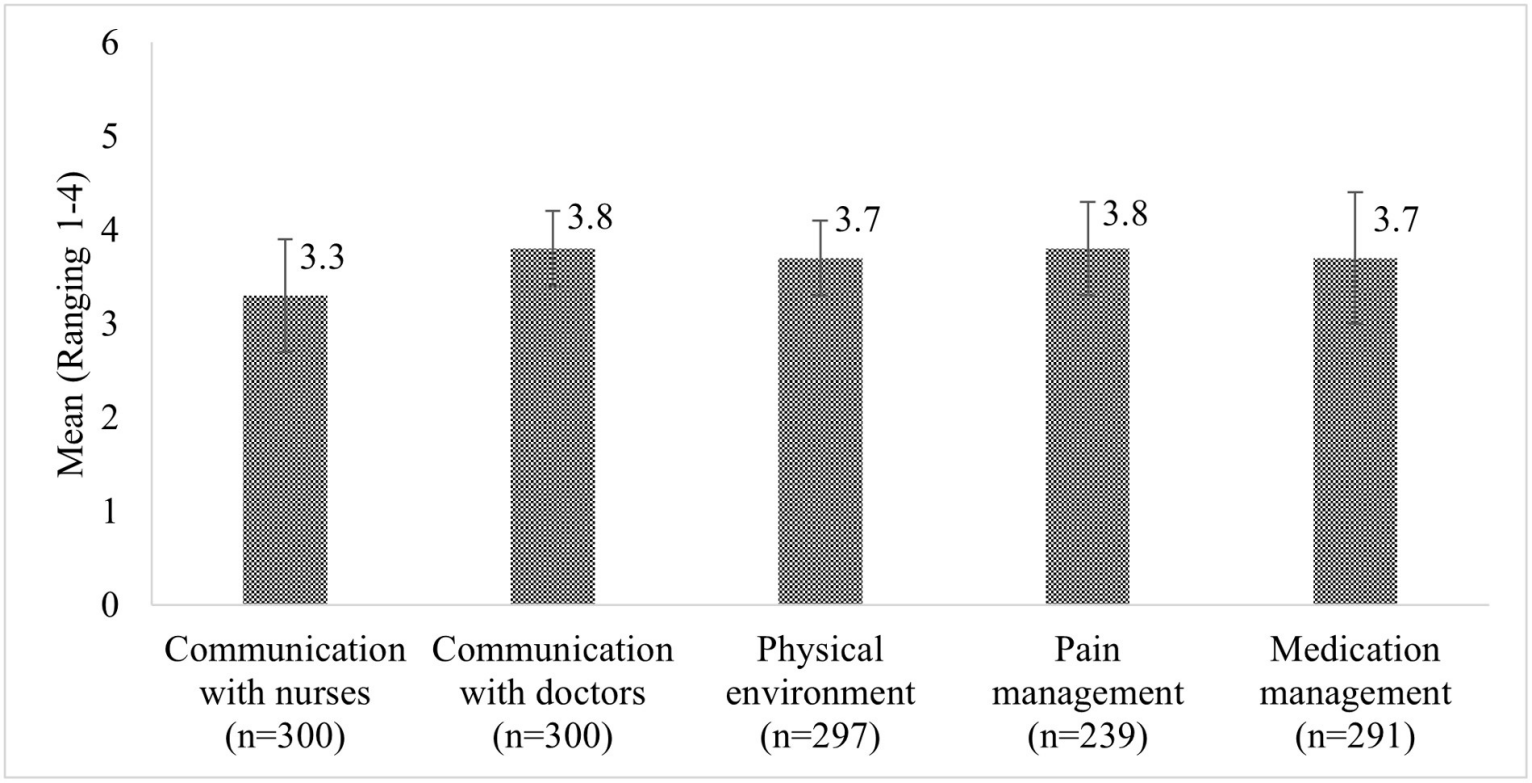
Inpatient experiences with service utilization (mean±SD).

The mean patient satisfaction with care quality was 8.82 out of 10 (SD = 1.07), with 28.7% rating services at 10. More than two-thirds (68.6%) rated the quality of care from 9–10 points and almost all patients (98.0%) expressed willingness to recommend the care facility to their family and friends (Table 2). Patients’ satisfaction with service utilization were presented in Fig 1. Patients had the highest mean satisfaction on communication with doctors and pain management at 3.8 out of 4 (SD = 0.4 and 0.5, respectively), followed by the physical environment and education about rationale for medication management (mean 3.7, SD = 0.4). Patients reported the lowest satisfaction on communication with nurses with a mean of 3.3 (SD = 0.6).

### Factors associated with overall satisfaction with oncological care

Three out of four I-PAHC domains were positively associated with the overall satisfaction with oncological care in the multivariable linear regression model (Table 3). Every additional unit increase in communication with doctors was associated with increased score of overall satisfaction ratings by 0.40 points (95% CI: 0.11, 0.70). Overall satisfaction scores were 0.33 (95% CI: 0.13, 0.53) and 0.30 (95% CI: 0.14, 0.45) units higher for an additional unit increase in communication with nurses and education about rationale for medication management, respectively. While women reported significantly higher than men (β = 0.29, 95% CI: 0.04, 0.53), patients experiencing delays in treatment scheduling observed 0.38 units lower for satisfaction with oncological care relative to those without any delays (95% CI: -0.64, -0.13).

**Table 3:** Factors associated with overall satisfaction ratings of oncological care in Vietnam: 2022.

	Coefficient	95% CI
Age in years	0.004	-0.002, 0.01
Being female (vs. male)	0.29*	0.04, 0.53
Chemotherapy department (vs. Radiation department)	0.04	-0.23, 0.3
First treatment at the study hospital (vs. no)	0.01	-0.3, 0.32
Delays in treatment scheduling (vs. no)	-0.38**	-0.64, -0.13
Communication with nurses (1–4)	0.33**	0.13, 0.53
Communication with doctors (1–4)	0.40**	0.11, 0.70
Physical environment (1–4)	0.13	-0.14, 0.39
Oncological medication management (1–4)	0.30***	0.14, 0.45
WHODAS functional impairment (12–60)	-0.005	-0.02, 0.01

*p<0.05;

**p<0.01;

***p<0.001.

## Barriers to accessing high-quality cancer care

Within the health system barriers domain, spending too much time waiting for an appointment (both delays in scheduling appointments and long waits for care at appointment times) was identified as a barrier by 28.8% of respondents (Table 4). Further, 24.1% reported a lack of coordination among service providers, followed by inadequate health insurance (13.7%). At the social/environmental level, 36.7% reported struggling to afford treatment and 36.7% reported facing transportation problems. Less than 10% considered the following to be barriers: cultural barriers (8.0%), communication issues with medical staff (5.3%), and social/financial support (2.3%). Among individual barriers, wages lost due to attending a medical appointment emerged as the most significant barrier to quality oncological care (43.3%). Over a third (36.7%) of respondents stated that fear, depression, anxiety, or distress were barriers. These were followed by limited knowledge about cancer and treatment (27.0%), competing life commitments (19.7%), and lack of information about where to find appropriate care (18.7%). Only three cancer patients (1.0%) reported an unwillingness to participate in healthcare decisions.

**Table 4:** Barriers to accessing cancer care in Vietnam: 2022.

	Total
	Total n (%)
**Health system barriers**	
Inadequate health insurance	41 (13.7)
Lack of coordination among service providers	72 (24.1)
Too much time waiting for an appointment	86 (28.8)
**Social/Environment barriers**	
Inability to pay for treatment-related expenses	110 (36.7)
Transportation problems	110 (36.7)
Social/financial support^a^	4 (2.3)
Communication issues with medical personnel^a^	16 (5.3)
Cultural barriers (e.g., believing in the supernatural method of treatment)	24 (8.0)
**Individual barriers**	
Fear, depression, anxiety, or distress	110 (36.8)
Worries that work wages will be lost to attend a medical appointment	52 (43.3)
Lack of information or understanding about cancer and its treatment	81 (27.0)
Denying or ignoring physical pain or symptoms	33 (11.0)
Lack of information about where to find appropriate care	56 (18.7)
Other health problems that make it difficult to get to the doctor or hospital	41 (13.7)
Being too busy with other life responsibilities (e.g., careers and school)	59 (19.7)
Unwillingness to participate in health care decisions	3 (1.0)

^**a**^Two items were modified compared to the original scale. Percentages of those who answered “somewhat of a barrier” or “a major barrier” were reported.

## Discussion

This study is among the first examinations of cancer patient experience at the tertiary-level oncological hospital at the national level, where the largest number of cancer patients receive treatment. Our findings indicated high overall satisfaction with the oncological care quality, aligning with a prior provincial-level study [14]. Similarly, our findings are also congruent with a study conducted in China, which showed that 30.0% of cancer patients rated satisfaction with oncological care at the highest level of 10 [17]—however, this study focused on rural areas, while our study recruited patients regardless of their residence. Additionally, most patients in this study expressed a willingness to recommend the study hospital to their families and friends, indicating high levels of satisfaction and trust in their oncological care. This finding concurs with prior data suggesting that cancer patients in China who received care at the higher, above county-level hospital within provinces were more likely to recommend their facility to friends and family compared to those who were at lower, county-level hospitals [17]. Cancer treatment is a long-term process, and therefore sustaining high patient satisfaction with care quality is critical for morale, attrition, and clinical outcomes.

We found that three out of four factors assessed by the I-PAHC questionnaire were positively associated with overall quality of care ratings. Notably, strong endorsement of positive communication with doctors and nurses emerged as the most consistent and impactful factors, suggesting that patient-centered communication strategies may be targeted to enhance the overall inpatient experience. Previous studies illustrate that good physician-patient communication plays a key role in increasing patient compliance with treatment strategies and patient satisfaction scores [20,21]. While the magnitude of association between the nurse-patient relationship and overall satisfaction may not significantly differ from the doctor-patient relationship (β = 0.33 and 0.40, respectively), it is still crucial to examine any potential barriers or challenges specific to nursing practice. This is attributed to the pivotal role nurses play in cultivating meaningful relationships with patients and their families, surpassing that of other members of the medical team [22]. By conducting such exploration, researchers and healthcare professionals can develop targeted strategies to enhance nurses’ communication skills, thereby leading to improved patient care. Likewise, educating patients about the purposes and side-effects of medication prior to administration was also strongly associated with patients’ overall satisfaction with their inpatient experience in our study. As patient-centered care is becoming an established practice in health care, it is increasingly the norm to involve patients in medical decision-making [23]. Policies and practices promoting enhanced provider-patient communication—including providing rationale for the introduction of new medications, and their risks and benefits—are key to improving patient experience.

In this study, physical environment was not significantly associated with overall satisfaction with healthcare, in contrast to prior findings in China [17]. This divergence may be explained by study settings, as our study included both rural and urban patients, while the Chinese study enrolled 443 rural patients [17], which may lack infrastructure to maintain the physical facility. Interestingly, our analysis revealed no significant association between functional impairment across six domains (life activities, cognition, self-care, mobility, getting along, and participation) and overall cancer care satisfaction. More research is needed to examine impact of each specific functional impairment domain on overall rating of quality cancer care in LMICs. Further, our final model excluded the pain management domain because only 79.7% (239/300) of patients reported pain-related issues and there was insufficient power to include this domain in the multivariable regression analysis. However, no substantial differences were observed between multivariable models with and without pain management. A previous study showed an insignificant association between pain management and overall ratings of healthcare satisfaction [17]. Future studies with larger patient cohorts are needed to examine the association between pain management and overall satisfaction with cancer care quality. Furthermore, considering the limitations of our quantitative approach, future studies employing a qualitative design could provide valuable insights by exploring the underlying narratives and lived experiences related to overall satisfaction with oncological care. Such an approach would enable a deeper understanding of the complex factors affecting satisfaction with cancer care in our specific context.

Oncological care satisfaction and barriers varied by demographics. Women were more likely to report high satisfaction with their cancer care compared to men, whereas those who experienced delays in treatment scheduling were less likely to report satisfaction with care quality. These findings were consistent with previous studies [24–26]. Among health system barriers, delays in appointment scheduling for treatment were the most frequently endorsed barrier to cancer care satisfaction. This is due to the high demand for services at specialized healthcare facilities, which results in patient dissatisfaction. Evaluation of factors contributing to scheduling delays is a critical next step in increasing patient satisfaction with oncological treatment. All patients in this study were insured and health insurance covered a mean of 86.8% of treatment expenses, yet 13.7% considered inadequate health insurance a barrier to accessing oncological treatment. Thus, the financial burden of treatment is a key barrier to satisfaction with cancer care [18]. However, the study population’s monthly income ($173.9) was significantly lower than Vietnam’s general population ($269.6) [27] and the median distance from the patients’ homes to the hospital was 62.1 miles. Thus, both financial and transportation constraints ranked high among social/environmental barriers in this sample, about a third (36.7%) of patients reported being unable to pay for treatment-related expenses, with the same number (36.7%) reporting transportation problems that lead to delays in treatment. This finding highlights the importance of task-shifting for point-of-cancer care from large, specialized urban hospitals to generalized, local healthcare facilities (e.g., province and district facilities) via provision of education about oncological care to providers at local facilities. This strategy could reduce the demand for specialized care as well as travel-related costs for patients and caregivers.

The most individual barriers to cancer care access was loss of wages due to attending a treatment. Additionally, the negative affect (fear, depression, anxiety, or distress) that often accompanies cancer were endorsed as barriers to cancer care access. Our previous study conducted at the same hospital found a high prevalence of mental health concerns, with 46.3% and 27.0% of patients exhibiting some depression and anxiety symptomatology, respectively [15]. Another prior work demonstrates that patients’ fears, anxieties and family members’ coping issues represent the most significant obstacles to care access [28]. Thus, to aid patients with psychosocial distress and increase satisfaction with treatment, evidence-based strategies such as mental health service provision and peer support during the cancer treatment phase may be indicated [29]. This study also showed that 27.0% of our sample endorsed limited knowledge on cancer and its treatment as well as guidance on finding appropriate care. Given the high rates of onset of cancer diagnoses, these results point to the importance of increasing availability of educational materials about cancer, cancer treatment, and available facilities for oncological care. Well-informed patients demonstrated greater capacity for treatment decision-making, adherence to treatment plans, and improved social function (e.g., ability to perform in daily life, leisure pursuits, etc.), and vitality (e.g., energy levels and reduced fatigue) [30–32]. Thus, informing patients is central to patient-centered care, as it increases patients’ capacity to actively partner with their treatment teams, take ownership of their treatment processes, and improve outcomes of cancer care.

Our findings should be interpreted with some limitations. Face-to-face surveys in treatment rooms may be influenced by social desirability bias but we minimized bias through rigorous methods, including training for data collectors, a pilot study, using show cards, and interviews without providers or family. Secondly, the sample, drawn only from the radiotherapy and chemotherapy units, was not fully representative, requiring further research to explore overall satisfaction immediately following biopsy diagnosis. Additionally, using a binary variable for treatment scheduling delays and unvalidated scales In Vietnam (e.g., I-PAHC and barriers to cancer care) could present a confound. Although Cronbach’s Alpha ensured the standardized scales’ internal consistency, further factor analysis is necessary to confirm their construct validity within the Vietnamese population. Additionally, the study’s cross-sectional nature restricts the establishment of causal relationships of overall oncological satisfaction. Future longitudinal studies would be instrumental in elucidating causal inference. Finally, while the overall quality of oncological care ratings was ordinal, a linear regression model was chosen based on a prior study’s recommendation [17] and for its comparative simplicity in interpretation. Subsequent analyses should consider implementing an ordinal linear regression model or utilizing a cut-off point for satisfaction to enable the use of a logistic regression model.

## Conclusion

Cancer patients in a large specialized oncological treatment center in Vietnam reported a high overall satisfaction with their care and indicated a strong inclination to recommend the facility to others. Despite the lack of scale validity, our findings also emphasize the importance of prioritizing patient-centered communication, increasing awareness of oncological medication, and mitigating treatment schedule delays to enhance patient satisfaction. Raising cancer patients’ understanding of cancer, cancer treatments, available care resources, and integrating mental health care into oncological services is a critical step to overcoming obstacles to accessing high-quality cancer care. Further research should recruit patients in outpatient services in order to comprehensively map patient journeys across the spectrum of services offered in oncological care to better understand factors that impact the care process across the range of care offered to cancer patients.
